# On the Pathology, Symptoms, and Treatment of Ulcer of the Stomach; with an Appendix of Cases

**Published:** 1858-11

**Authors:** 


Art. IV.—*On the Pathology, Symptoms, and Treatment of Ulcer of the Stomach; with an Appendix of Cases.* By Wm. Brinton, M.D., Fellow of the Royal College of Physicians, Physician to the Royal Free Hospital, Lecturer on Physiology and Forensic Medicine in St. Thomas's Hospital. 8vo., pp. 227. London: Churchill, 1857.

The above work is a reprint, with some alterations and additions, of various papers on ulcer of the stomach, recently contributed by Dr. Brinton to the *British and Foreign Medico-Chirurgical Review,* the *Lancet,* and the *Association Journal.*

As many of our readers have probably already become acquainted with parts of this excellent treatise through the periodical press, we will limit ourselves to the consideration of the most striking points and questions connected with the subject of the author's inquiry. Dr. Brinton states in the preface, in allusion to the plan upon which he has conducted his researches, that wishing to make his inquiry a study from nature, he has drawn his materials exclusively from cases, either observed by himself or recorded by others. The fact that he has investigated a greater number of cases of gastric ulcer than any previous writer on the same subject, adds considerable weight to his opinions, and entitles his treatise to the particular attention of the profession.

The *first part* of Dr. Brinton's work treats of the *Pathology* of Ulcer of the Stomach, and contains all the information on this point which could be deduced from extensive and elaborate statistical researches. That numerical calculations made from the results not only of his own observations, but of about eleven hundred cases gathered from various journals, reports, and museums, would lead often to mere conjectures, the author himself readily admits. He sometimes, however, loses sight of this circumstance in the course of his investigations, and uses these conjectural deductions as positive facts; as, for instance, in establishing the etiology of perforating ulcer. We give in the following a very condensed abstract of the results Dr. Brinton has obtained from his researches, referring our readers for the details and numerical data to the monograph itself.

Evidence of the present or previous existence of gastric ulcer may be found in from 2 to 13 per cent. of persons dying from all causes; and the ulcer itself, open and unhealed, may be observed in from 1 to 10 per cent. The 7226 collected necropsies offer about 360 ulcers, which are pretty equally divided into 190 open ulcers, and about 170 cicatrices. These numbers nearly correspond to a total proportion of 5 per cent., which is divisible into $2\frac{3}{5}$ and $2\frac{2}{5}$ per cent., for these two conditions respectively. It is to be remarked, that the maximum frequency of the ulcer occurs in the spirit-drinking population of Copenhagen; and that its larger proportion in the German hospitals may be plausibly referred to the inmates of these institutions being, on an average, of greater age, if not in more destitute circumstances, than the persons annually received into English hospitals.

Ulcer of the stomach is twice as frequent in the female as in the male, and seems to be especially, though not exclusively, a disease of middle and advancing life. The posterior surface of the stomach, the lesser curvature, and the pyloric pouch, seem to be more liable to ulcer than the greater curvature and the cardiac sac. A plurality of ulcers in the same stomach was found in about 21 per cent. The shape of this lesion is usually circular or slightly oval, but is liable to some variation. The tissues around the margin of the ulcer may present every variety of appearance; in some instances there may be almost a complete absence of inflammation; in others, signs of inflammation may exist, associated with thickening, presenting the appearance of cancer. The base of the ulcer resembles the margin, and may be either firm and hard, or soft and flocculent. In progressing in depth, the ulcer forms a cone, with its apex directed toward the peritoneum, and results either in perforation or in adhesion of the stomach to some neighboring viscus; the latter mode of termination is found in about 40 per cent. of the cases. The disease is of very variable duration; in exceptional cases it has proved fatal in as few as ten days, by perforation; by exhaustion caused or hastened by vomiting; rarely by hemorrhage; generally, however, it lasts from several weeks or months to many years. Dr. Brinton records cases of 35, 30, and 20 years' duration. Cicatrization of the ulcer is about as frequently met with as the ulcer itself; in other words, half the instances of this disease undergo what is probably a spontaneous cure. Where the ulcer has been very large, its cicatrix frequently corresponds to a constriction of the organ, amounting in extreme cases to an absolute stricture impeding the transit of food, and thus causing gradual hypertrophy and dilatation of the over-distended segments of the stomach behind the constriction. Perforation seems to be an exceptional occurrence in gastric ulcer; the proportion of its frequency amounts to about 13·4 per cent. Quite in contrast with the simple ulcer, which especially affects middle and advancing life, and becomes more frequent in proportion to the age, is the perforating ulcer, which selects an earlier period of life, and the liability to its occurrence decreases as life advances. It is worthy of especial notice, that the greatest number of cases of perforation in the female occur between the ages of fourteen and thirty, while nearly two-thirds of that number are found between the ages of fourteen and twenty. As regards the situation of perforating ulcer, statistical calculations seem to prove that the anterior surface of the stomach, though more rarely ulcerated than the posterior, is more liable to perforation. The consequences of perforation are general peritonitis, proving fatal, or circumscribed peritonitis, with the formation of abscesses and adhesions to the abdominal wall or diaphragm, and consequent effusion externally, or into the cavity of the chest. The communication of the stomach with other parts of the alimentary canal, as the result of gastric ulcer, is generally independent of any such abscesses. It frequently becomes adherent to the liver and pancreas; more rarely to the spleen; the dangers of such adhesions are hemorrhage from an erosion of the vessels of these organs, and gangrene of the liver or spleen. The frequency of death from hemorrhage, in proportion to the cases of ulcer, is stated differently at 5, and at $3\frac{1}{4}$ per cent.; the latter average being obtained from a large number of cases, collected chiefly from British sources. In 52 of these cases the sex is mentioned—34 being male, and 18 female. As ulcer occurs twice as frequently in the female as in the male, the liability to a fatal termination by hemorrhage seems to be four times greater in the latter than in the former. In 34 cases the source of hemorrhage was in three instances the substance of the liver, in one instance the spleen, and in the remaining 30 cases a large vessel of the stomach, or of its immediate neighborhood; in sixteen of the twenty-nine instances in which the vessel itself can be named, the hemorrhage was the result of an ulcer which eroded the splenic artery in its course along the upper border of the pancreas. Concerning exhaustion or starvation, which forms another of the fatal terminations of gastric ulcer, statistical information could not be obtained. Of about 14 cases, however, collected by the author, seven probably died from exhaustion, in consequence of vomiting of food. Dittrich found three in 103 cases, in which death was caused by tabes referable solely to the ulcer. As respects the combination of gastric ulcer with cancer of the stomach, it is often limited to a cancerous degeneration or deposit that involves the brawny mass which is generally present in the base or periphery of an ulcer of long standing. The most frequent form, however, appears to be that in which a fungus, generally a bleeding one, shoots up from the base of a gastric ulcer. Lastly, in regard to the complications of gastric ulcer with other diseases, such as phthisis, pneumonia, diarrhœa, dysentery, and syphilis, the author judiciously remarks, that they merely seem to indicate that this long and exhausting malady predisposes the constitution to a variety of other diseases, and renders unusually fatal many of those attacks of illness which in the course of years very few persons altogether escape. This is the only probable, though vague, relation that seems to exist between gastric ulcer and disease generally; a direct causative influence between this disease and any special malady, we are by no means justified in assuming.

Finishing the first part of his work, the author illustrates how much we have yet to learn respecting even the more obvious pathological relations of gastric ulcer by the following summary: "Let us suppose that of every 100 ulcers of the stomach 50 cicatrize, $13\frac{1}{2}$ perforate its walls, $3\frac{1}{4}$ erode its larger vessels, and 2 or 3 kill by the sheer exhaustion and inanition they involve, we have still a proportion of about 30 ulcers in every 100 left, quite unaccounted for. In other words, we have yet to determine the termination of nearly one-third of all the instances of this lesion."

In the *second part* of his treatise the author subjects the *symptoms* to a careful and accurate analysis. To render his statements as unbiased and reliable as possible, he has compared the results of his own extensive experience with the records of several hundreds of cases, verified by careful necropsy. His monograph thus contains a complete *résumé* of the present state of our knowledge on the semeiology of ulcer of the stomach.

In describing the different symptoms individually, Dr. Brinton dwells first upon *pain* as the most frequent and constant of all. The effect of pressure is, in his opinion, a "very important test." He demands, therefore, that in order to diagnosticate "ulcer," the seat and severity of the pain should be determined by applying pressure to the epigastrium, which generally will increase both the epigastric and spinal pain; instances in which the pain is diminished on pressure are very rare; that the time when the dorsal pain was first observed, as well as its situation, should be carefully noticed, and compared with those of the epigastric pain; that the influence of movements of the body, and of the decubitus upon the pain should be studied; Dr. Brinton found that in ulcer on the anterior surface of the stomach the pain is diminished by the supine decubitus, on the posterior surface by the prone; and finally, that the effect of various kinds of food upon the pain should be taken into consideration.

In spite of the very minute description of the different characteristics of this one symptom, we think that the author has been guilty of exaggeration of its value. Increase of epigastric and dorsal pain on pressure would indicate merely that the gastric mucous membrane is in an inflamed or otherwise irritated condition, and can, therefore, not be considered a characteristic sign of ulcer. That the pain of ulcer is frequently affected by changes in the state of abdominal innervation the author himself shows conclusively, by pointing out the connection between the pain and the menstrual molimen.

*Vomiting*, which forms the next symptom of gastric ulcer, exhibits far fewer peculiar characters than the pain. Whether the seat and size of the ulcer exercise any influence upon the frequency of this symptom could not be decided with any degree of satisfaction.

The next symptom the author subjects to his analysis is *hemorrhage.* If merely of capillary origin, this symptom is rarely discovered. In a third of the cases in which hæmatemesis and melæna is observed, the hemorrhage originates, according to the author's observation, from the larger vessels of the sub-mucous or subserous areolar tissue, or from those distributed to the liver, spleen, and pancreas. This hemorrhage may be so profuse as to distend quickly the stomach and intestines, and to produce death by syncope. Here again the author omits to point out that the symptom under consideration is by no means characteristic of ulcer of the stomach; even a profuse hemorrhage, accompanied by all the other symptoms of the disease, would not justify us in assuming the existence of an ulcer, unless all the other sources of gastric hemorrhage, as disease of the heart, cirrhosis of the liver, obstructions in the portal circulation, purpura, etc., could be excluded. Two other sources of gastric hemorrhage he enumerates under the head of ætiology of gastric ulcer; we refer to "ague" and "hemorrhagic erosions." Following the view of Rokitansky, most pathologists believe that these erosions, the cause of which is still unknown, merge into ulcer. Dr. Brinton opposes this view on the ground of post-mortem appearances, and of the different train of symptoms observed in the two diseases.

In proceeding in his analysis of the symptoms of ulcer, the author dwells at length upon one, observed frequently in cases of the disease in the female, viz.: *amenorrhœa.* Here he shows by his statistics, that menstruation is generally normal in cases of ulcer, and it is only after considerable hemorrhage from the ulcer that amenorrhœa sets in, and that the occurrence of so-called "vicarious menstruation," in the form of periodic hæmatemesis, is not at all positively proved. The case recorded by Cruveilhier is based solely on the testimony of the patient, given many years after it had been observed. On the other hand, he dwells upon the simultaneous occurrence of amenorrhœa and perforation, which is so frequently observed, and was first pointed out by Crisp, and tries to bring both in direct relation.

According to his statistical investigations of cases which ended fatally by perforation, in the female, the greatest number is observed among young girls who are not yet fully developed, are approaching, or have just passed the age of puberty, and suffer from anæmia, or scanty and irregular menstruation. This circumstance would naturally suggest the question, whether the amenorrhœa causes the ulcer, or the ulcer the amenorrhœa. The first part of this proposition the author answers in the negative, "not only because the ulcer is present in the male sex, and in the neuter monster, at the same age, as well as in the female at all other ages, but because in the female, at this epoch of life, the exceptions to the presence of the supposed cause are too numerous to be compatible with such a causation." He is in favor of an affirmative answer to the second part of the question, and the reasons he gives are perfectly tenable. The dyspeptic symptoms, which correspond with the establishment of the lesion, generally precede the deficiency or cessation of menstruation, and develope an anæmic condition, the usual attendant of all cachexias. Where, however, a cachexia is being developed, there the tonicity of the solid parts is already changed and lowered; and in the same measure in which the force and energy of these parts is diminished will not only their functions deviate from the normal standard, but also will the nutrition of the tissues be morbidly changed.

Although the usual results of a cachexia may be well known to us, its ultimate cause is not so obvious, since we are ignorant of the power which forms and maintains the tissues. Dr. Brinton is, therefore, perfectly right in maintaining, in his elaborate discussion on *the ætiology* of gastric ulcer, that there is nothing specific in this disease. He believes, on the contrary, that all the varieties that affect the form, progress, situation, numbers, and terminations of this lesion, find their parallel in the causation of the malady, which he refers to a certain cachectic condition of the system, the essential nature of which is as unknown to us as that of cachexias in general; in short, that we have no more right to speak of *the* ulcer of the stomach than of *the* ulcer of the leg, either of which lesions may commence in a great variety of ways, viz.: by ecchymosis, softening, a varicose vein, etc. He quotes Mr. Critchett's experience in reference to the character of the ordinary cutaneous ulcer, occurring at the epoch immediately following the access of puberty, and points out the analogy between it and the gastric ulcer at the same period, especially in regard to one peculiarity,—the more or less complete absence of inflammatory reaction at the base and margins. This process of *destructive absorption* is observed in another very striking instance, not mentioned by Dr. Brinton; we refer to the exfoliation and ulceration of the cornea following sometimes an unduly prolonged period of lactation, or originating spontaneously in the course of typhus, and of hydrocephalus. This marked example of ulceration occurring in a tissue in a primary manner, uncomplicated often with any other morbid action, is, according to the observations of Dr. Handfield Jones, always associated with states of debility, and is most benefited by a tonic and restorative treatment.

Under the head of *diagnosis*, Dr. Brinton lays down the following as the minimum of evidence that will justify our affirming the existence of an ulcer of the stomach during life: The pain must possess the characters attributed to it, must be accompanied by vomiting, and there must be evidence of hemorrhage having occurred in the course of the malady. He concedes, however, that in a large number of cases in which we may justifiably suspect the presence of an ulcer, some of these symptoms may be absent, in which event an absolute enforcement of the above rule of diagnosis might be the occasion of grievous errors in practice. In cases, for instance, of severe or protracted dyspepsia, associated with gastric pain, which is especially called forth by proteinous substances, or by hot liquids, and is affected by movement, rest, and posture,—particularly if such symptoms occur in connection with amenorrhœa in young females about the period of puberty,—Dr. Brinton deems it advisable that we should keep the possibility of gastric ulcer steadily in view; indeed, by treating such cases as ulcer of the stomach, we may often cure what we cannot diagnose. In regard to the diagnostic value of one of the symptoms enumerated above, gastric hemorrhage, it is to be mentioned, that its occurrence in a moderate form would not indicate invariably the existence of an ulcer, as it may take place without a "breach of continuity" in the gastric mucous membrane. Only in cases of more profuse hemorrhage in which the causes mentioned in an earlier part of our review could be excluded, could the existence of an ulcer be diagnosed with any degree of certainty.

In the *treatment* of gastric ulcer Dr. Brinton lays down the following indications: to remove all local obstacles to the cicatrization of the ulcer; to support the constitution in effecting this process; to remedy the results the lesion may have already brought about; and to limit or arrest some of the more prominent symptoms by which these results are usually betrayed.

To meet the first two indications, the author considers a strict regulation of diet of far greater importance than the exhibition of drugs, which he regards merely as adjuvants. The diet should consist at first of milk, given in small quantities, and at frequent intervals; if the irritability of the stomach is more moderate, farinaceous substances, the most suitable of which is arrow-root, may be added; and finally, as convalescence proceeds, animal diet may be cautiously tried. With reference to the last two indications, the author discusses the value and applicability of the different remedies usually employed for the purpose. He recommends especially, *opium* as a means of promoting the cure of ulceration, and justly praises its value in ulcers of long duration, of obstinate character, and in broken, exhausted constitutions, as it will not merely relieve pain, check great irritation or undue secretion of the mucous membrane, but will also support the strength, buoy up the nervous system, and check the waste or expenditure of the tissues generally. During convalescence the author urges the necessity of great caution in returning to the ordinary diet, as an excess at the table may suddenly bring back all the symptoms of the disease, even months after their disappearance. Patients should, therefore, for a long time after their recovery, take food only in small quantities; have this well prepared, not swallow it too hot, and only after thorough mastication.

The *appendix* to the treatise contains a series of reports, published originally in the "Association Journal." They are a selection from numerous cases of ulcer of the stomach, observed and described by the author in a very complete and accurate manner, and will be found, on perusal, to be very good illustrations of the more common and important varieties of the malady.

